# Stress Cardiomyopathy in the Setting of COPD Exacerbation

**DOI:** 10.1177/2324709615612847

**Published:** 2015-10-14

**Authors:** Kevin Landefeld, Qusai Saleh, Gary E. Sander

**Affiliations:** 1 Tulane University School of Medicine, New Orleans, LA, USA

**Keywords:** stress cardiomyopathy, takotsubo cardiomyopathy, β_2_-agonists, COPD

## Abstract

*Introduction*. Stress cardiomyopathy, or takotsubo cardiomyopathy, is an acute, reversible left ventricular dysfunction usually initiated by a psychological or physical stress. We report this case of stress cardiomyopathy following a chronic obstructive pulmonary disease exacerbation and the subsequent treatment. *Case Description*. A 49-year-old white female with a history of chronic obstructive pulmonary disease presented to the emergency room via emergency medical services with worsening severe shortness of breath and productive cough for 2 weeks but denied any chest pain on arrival. On presentation, she was noted to be tachypneic, using her accessory muscles and with bilateral coarse expiratory wheezing on lung auscultation. Initial electrocardiogram demonstrated sinus tachycardia. She was treated with multiple albuterol treatments. Soon afterwards, the course was complicated by hypoxic respiratory failure eventually requiring intubation. Her repeat electrocardiogram showed acute changes consistent with myocardial infarction, and an echocardiograph demonstrated apical akinesia with an ejection fraction of 25% to 30%. The patient was urgently taken for cardiac catheterization, which showed no angiographic evidence of coronary artery disease. Three days after initial presentation, a repeat transthoracic echocardiogram showed overall left ventricular systolic function improvement. *Discussion*. This case provided a unique look at the difficulty of balancing catecholamines in a patient with bronchospasm and stress cardiomyopathy.

Stress cardiomyopathy, also known as takotsubo cardiomyopathy, is an acute reversible left ventricular dysfunction that is typically provoked by psychological or physical stress. The clinical presentation, though variable, can be one with electrocardiographic findings mimicking acute myocardial infarction, but with no evidence of epicardial coronary artery disease on angiography.^1^ It is most commonly characterized by abnormal contractile motion of the apex of the left ventricle with relative sparing of the basal segments, but other variants have been reported.^2^ Less than 40 cases of stress cardiomyopathy induced by severe respiratory compromise due to chronic obstructive pulmonary disease (COPD) or asthma have been reported; this syndrome has occurred primarily in women (81.6%) with an age of approximately 65 years.^3^ We report the case of a 49-year-old woman with a severe COPD exacerbation and respiratory failure treated with short-acting β-adrenergic agonists leading to stress cardiomyopathy.

A 49-year-old white female with a history of COPD presented to the emergency room with worsening severe shortness of breath and productive cough for two weeks. She denied any chest pain on arrival. On presentation, she was noted to be tachypneic at 28 bpm, using her accessory muscles, and with bilateral coarse expiratory wheezing on lung auscultation. She was mildly hypoxic, and chest radiograph showed hyperinflation without evidence of pulmonary edema; electrocardiogram (ECG; Figure 1A) showed sinus tachycardia at a rate of 138 bpm. She was subsequently given nebulizer treatments with ipratropium bromide and albuterol sulfate followed by high-dose intravenous methylprednisolone. She had improvement in her oxygen saturation, but continued to have evidence of bronchospasm on physical examination and was started on two consecutive nebulizer treatments with albuterol sulfate in addition to intravenous aminophylline. Notably, her initial basic laboratory (complete blood count, basic metabolic panel) workup was unremarkable. Soon afterwards, the patient developed hypoxic respiratory failure requiring endotracheal intubation. At that time, ECG showed ST elevation consistent with inferoposterolateral myocardial injury (Figure 1B). Troponin I levels were mildly elevated with a value of 1.6 ng/mL (normal value, <0.04 ng/mL). Parasternal long axis and subcostal 4-chamber view on transthoracic echocardiogram showed left ventricular ejection fraction (LVEF) of 25% to 30% with apical and anterolateral walls akinesis (Figure 2). There was also evidence of severe pulmonary hypertension with pulmonary artery systolic pressure estimated at 100 mm Hg. The patient then developed hypotension and was started on vasopressors. She was urgently taken for cardiac catheterization, which showed no angiographic evidence of coronary artery disease (Figure 3). Three days after initial presentation, a repeat transthoracic echocardiogram showed LVEF had increased to 45% to 50%, with mild LV global hypokinesis. Subsequent ECGs demonstrated diffuse T-wave inversion and a prolonged QT interval (Figure 1C), a common finding in stress-induced cardiomyopathy patients. The patient continued to improve with respiratory treatments and oral steroids and was eventually discharged home.

**Figure 1.**
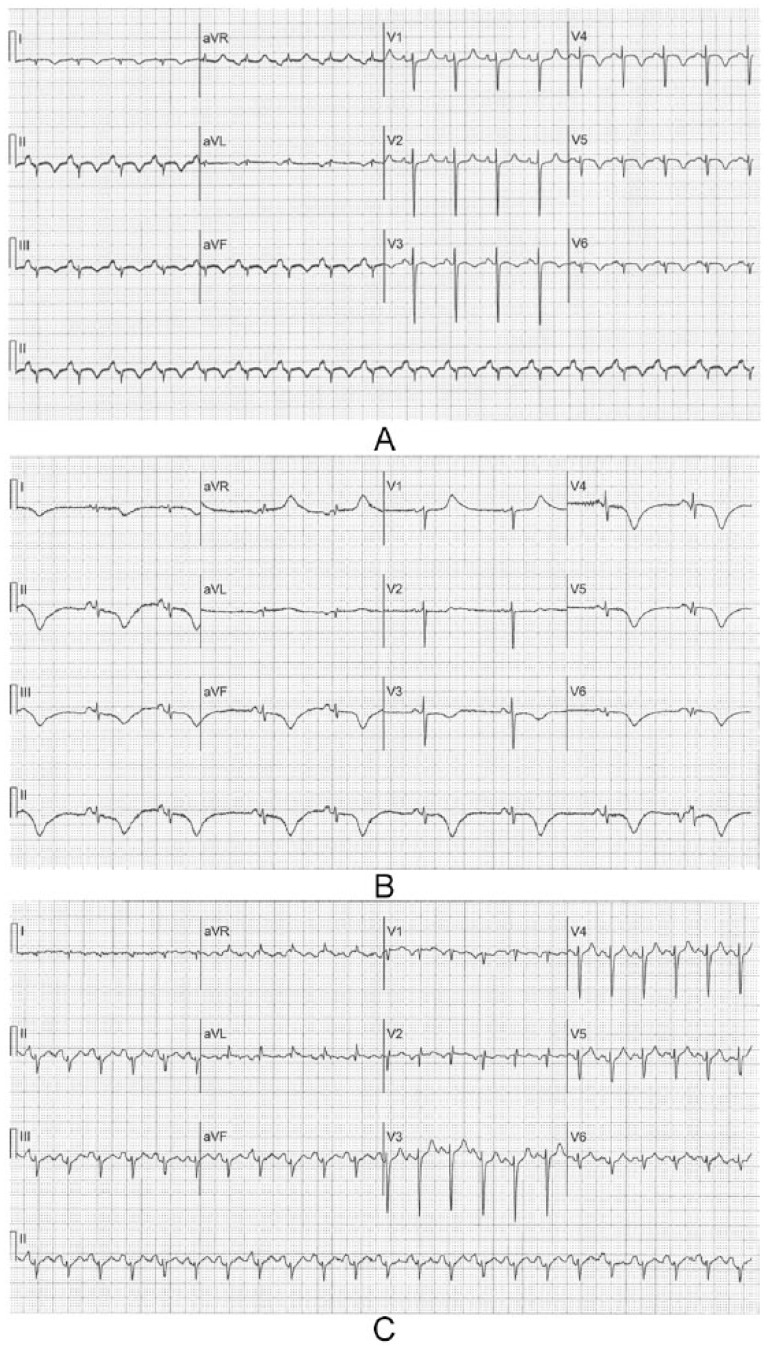
ECG tracings demonstrating (A) the initial ECG at the time of presentation with acute respiratory distress, (B) the changes consistent with acute myocardial injury recorded 24 hours later, and (C) subsequent ECG showing diffuse T inversions and prolonged QT interval.

**Figure 2.**
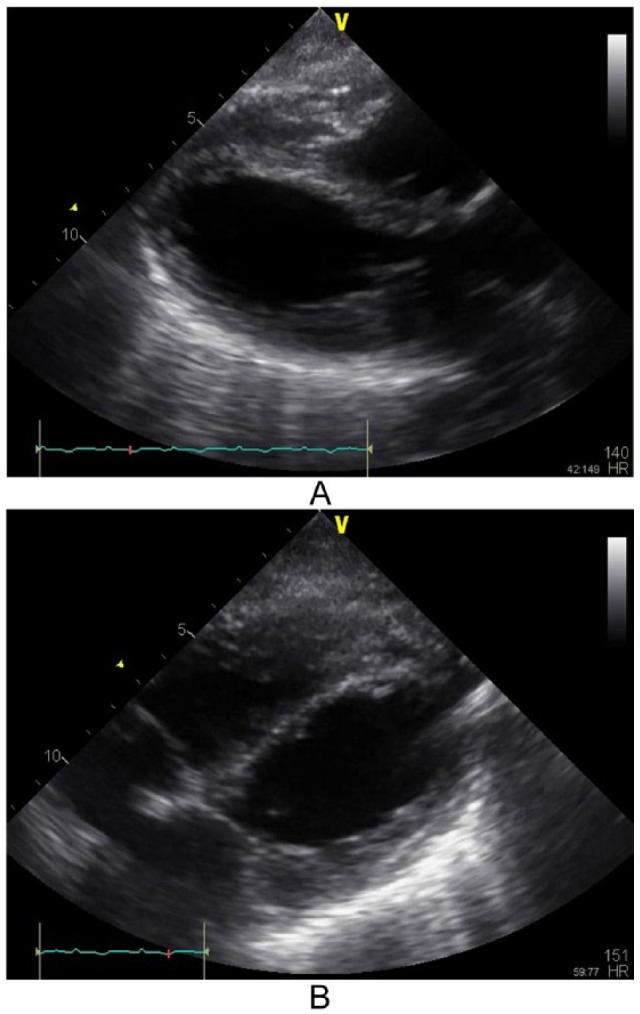
Transthoracic echocardiogram images showing left ventricle during peak systole demonstrating apical akinesis typical of stress cardiomyopathy: (A) parasternal long axis; (B) subcostal 4-chamber view.

**Figure 3.**
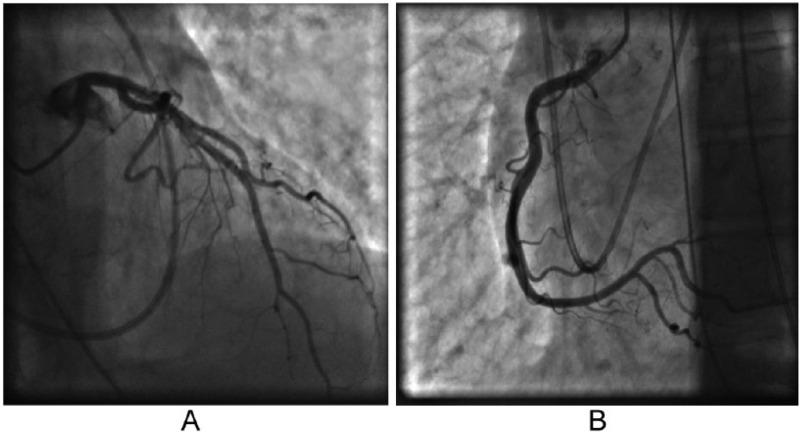
Coronary angiograms indicating normal coronary arteries.

The exact mechanism of stress cardiomyopathy remains unclear. However, several mechanisms have been suggested to explain the association between sympathetic stimulation and myocardial stunning.^4^ These include ischemia resulting from epicardial coronary arterial spasm due to increased sympathetic tone, microvascular spasm, and catecholamine-mediated myocardial stunning causing direct myocyte injury. It has been shown that elevated catecholamine levels decrease the viability of myocytes through cyclic AMP–mediated calcium overload resulting in contraction band necrosis, a histologic pattern of myocyte injury seen in stress cardiomyopathy.^4^ Troponin I assays have been found to be elevated in stress cardiomyopathy but not to the extent of acute myocardial infarction.^5^ The elevated enzymes indicate that a type II myocardial infarct has occurred, but it is unclear as to whether this is the result of reduced coronary flow or a direct toxic effect of myocytes. Abraham et al^2^ provided compelling evidence that exaggerated sympathetic stimulation after intravenous administration of catecholamines together with β-receptor agonists is sufficient to precipitate the stress cardiomyopathy in susceptible individuals, and they demonstrated different ballooning patterns suggesting a complex interaction between sympathetic innervation, myocardial β-receptor density, and catecholamine sensitivity. It has been suggested that an increase in β_1_/β_2_ adrenoceptor ratio in the apical ventricular region may explain the apical dysfunction of takotsubo cardiomyopathy.^6^ However, it has been reported that perhaps 20% of cases occur in patients taking β-blockers, and this observation suggests that alternative pathogenic mechanisms may be involved as suggested above.^1^ However, β-blockers are competitive inhibitors of β-adrenoceptors, and thus massive increases in circulating catecholamines may still result in receptor activation.

It is unclear whether the interval worsening of respiratory status in our patient was the initial trigger for a catecholamine surge causing the cardiomyopathy, or whether the use of repeated albuterol treatments may have triggered the stress cardiomyopathy. It has been previously demonstrated that high-sensitivity troponin assays can be elevated in 74% of patients admitted to hospital with an exacerbation of COPD.^7^ Stress cardiomyopathy may be relatively common in this setting, but is difficult to detect clinically without appropriate early cardiac investigation.

Treatment for COPD exacerbation may, therefore, be a distinct challenge. Traditional treatment with β_2_-agonists for bronchospasm may trigger or worsen left ventricular failure. Bronchospasm could be better treated using anticholinergic agents, steroids, and mast cell stabilizers, thus avoiding β-agonists. If a β-agonist were to be used, levalbuterol has been shown to be more pulmonary specific. Stress cardiomyopathy as a complication of severe COPD exacerbation should be considered, as treatment may need to be tailored to limit the use of β-agonists and treat acute left ventricular failure. Future studies are needed to further identify individuals at risk of stress cardiomyopathy with such presentations.
